# Impact of landfill leachate contamination on surface and groundwater of Bangladesh: a systematic review and possible public health risks assessment

**DOI:** 10.1007/s13201-021-01431-3

**Published:** 2021-05-29

**Authors:** Fahmida Parvin, Shafi M. Tareq

**Affiliations:** Hydrobiogeochemistry and Pollution Control Laboratory, Department of Environmental Sciences, Jahangirnagar University, Dhaka, 1342 Bangladesh

**Keywords:** Landfill, Leachate pollution index, Water contamination, Health risk assessment, Bangladesh

## Abstract

Dumping of solid waste in the non-engineered landfill is very common in the developing countries. Among the different disadvantages of this kind of landfilling, leachate is the major concern to public health, which is a toxic byproduct generated from the landfill; and can percolate to the ground water and consequently migrate in surface water. Using systematic review on published data, the present study endeavors to compare the leachate contamination potential of four major landfills of Bangladesh, named Amin Bazar, Matuail, Mogla Bazar and Rowfabad; which are situated in 3 of the 6 big mega cities of Bangladesh and assessed the effects of leachate leakage on surrounding water body as well as on human health. This study, for the first time calculated the leachate pollution index (LPI) for the landfill sites of Bangladesh and found that the LPI of Matuail landfill site (19.81) is much higher which is comparable to some polluted landfill sites of India and Malaysia. The concentrations of several potentially toxic metals found in the surface and ground water in the vicinity of the landfill sites were above the maximum permissible limit values of department of Environment, Bangladesh and World Health Organization (WHO). The human health risk index for toxic heavy metals in different vegetables and rice grain showed high health risk potential for Pb, Cd, Ni, and Mn. The total carcinogenic risk for Ni and Pb are found very high in the edible plants near those landfill sites, suggesting the risk of Ni and Pb induced carcinogenesis by the consumption of those plants. The present conditions of surface, ground water and agriculture products near the landfill sites of Bangladesh are much frightening to the biota and local inhabitants.

**Supplementary Information:**

The online version contains supplementary material available at 10.1007/s13201-021-01431-3.

## Introduction

Annual waste generation is increasing exponentially with rapid population growth, urbanization and industrial development in Bangladesh (Alam and Qiao 2020). The dumping of non-segregated solid waste to landfill sites is the most prevalent waste disposal practice in developing countries such as Bangladesh (Jahan et al. 2016; Kamal et al. 2016; Hossain et al. 2018; Xaypanya et al. 2018; Alam et al. 2020) and even in the part of developed countries (Mishra et al. 2019). The improper management of landfills and generation of toxic leachate thereby exert significant impacts on surrounding freshwater and groundwater (Toufexi et al. 2013; Kamal et al. 2016; Mishra et al. 2019). Leachate is the aqueous effluent generated from solid waste owing to their physical, chemical, and biological alteration in landfills (Youcai 2018) and is considered as a chemical soup of dissolved organic matter (DOM), xenobiotic organic compounds, different anions and cations, and heavy metals (Christensen et al. 2001). Among the different component of landfill leachate, the heavy metals are non-biodegradable, able to deteriorate the surface and groundwater quality and toxic even at low level to biological system (Fergusson 1991; Akpor 2014; Gautam et al. 2014; Verma 2017). Heavy metals are also persistent, bio-accumulative, and toxic as well as endocrine disrupting and carcinogenic (Kibria et al. 2016). Conversely, the DOM which constitute a large portion of leachate, has potential to bind with heavy metal, and consequently plays a significant role in the bioavailability of those metals in the aquatic environments (Baun and Christensen 2004; Rikta et al. 2018).

The principal concern about municipal landfill is focused on the pollution potential due to mobilization of the generated leachate through the subsoil into the surface and groundwater (Kjeldsen et al. 2002; Fadhullah et al. 2019; Mishra et al. 2019). Further, during the wet season, water containing leachate from landfill site drains into the nearby lowlands and surface water bodies and pollutes the local environments (Hossain et al. 2018). Hence, this toxic aqueous effluent from landfill site can causes potential risks to surface and groundwater (Christensen et al. 2001; Vaccari et al. 2019) and eventually found to poses a threat for aquatic biota, plant and public health (Toufexi et al. 2013). Iswa, (2013) reported that in most of the developing countries, improperly managed open landfill sites are more commonly practiced than controlled and engineered landfills. Residents, especially the urban and semi urban poor in those countries are affected severely by this uncontrolled management of waste via water and food contamination by toxic leachate.

Bangladesh, as an over populated country (164 million in 2020, www.Worldometers.info) generated around 8000 tons of solid waste each day (Abedin and Jahiruddin 2015) and disposed the solid waste in an uncontrolled manner (DNCC 2016). To the best of our knowledges, very few studies have conducted to assess systematically the contamination level of landfill leachate around different landfill sites in Bangladesh (Jahan et al. 2016; Kamal et al. 2016; Hossain et al. 2018; Alam et al. 2020). Moreover, none of the study analyzed properly the level of leachate pollution potential of landfill sites of Bangladesh and to which extent leachate contaminates the groundwater and surface water. Furthermore, the municipal water supply system of Bangladesh is dependent mostly on ground water (78%) and to some extent on surface water (22%) (Khan 2019). Hence, it's significant to understand the contribution of landfill leachate in polluting surface and groundwater of Bangladesh especially urban and sub-urban areas is urgently required.

Given the importance of the above issues, it is decisive to do more detailed systematic review and meta-analyses on leachate characteristics, especially the heavy metal and organic pollutants in sanitary landfills and dumpsites from different locations of Bangladesh; and the health risk of leachate, as well. Using available published data, this study reviewed the status of leachate pollution potential of four different major landfill sites of Bangladesh, namely Amin Bazar, Matuail, Mogla Bazar, and Rowfabad in the recent decade and the contribution of leachate to contaminate the water body in the vicinity of the landfill site. In addition, this study first time analyzed the leachate pollution of the landfill sites using leachate pollution Index (LPI) and compare the level with other neighboring countries. Furthermore, this study calculated the health risk of consuming the edible plants and fish grown near those landfill sites. Our study can aid the waste management authorities and landfill operator’s to understand the severity of the landfill leachate pollution in Bangladesh and to make appropriate preventative measures against surface /groundwater contamination.

## Materials and methods

### Strategy of search

**Fig. 1:**
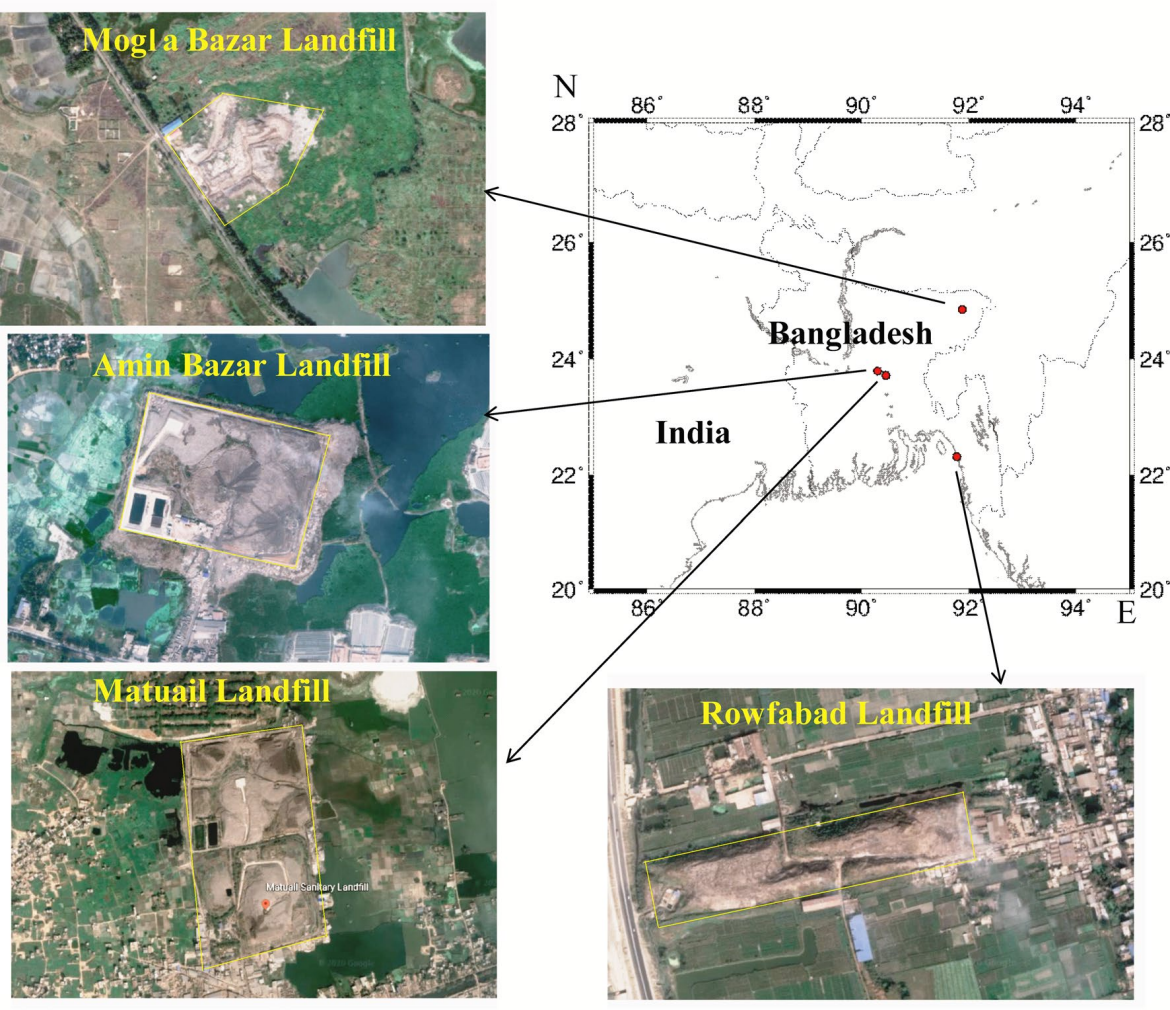
Map of the study areas in Bangladesh. The yellow marked area in the figures indicates the landfill sites

This study has been accomplished by utilizing the published data (Azim et al. 2011; Hossain et al. 2014, 2018; Jahan et al. 2016; Kamal et al. 2016; Alam et al. 2020) on landfill leachate of four landfill sites of Bangladesh named Amin Bazar, Matuail, Mogla Bazar, and Rowfabad (Fig. 1); which are situated in 3 of the 6 big mega cities of Bangladesh and contamination in water bodies and edible plants in the vicinity of those landfill sites. In order to retrieve the published scientific articles which are relevant to this work, a systematic search had been done in the publicly available databases (Science Direct, Scopus, PubMed, and Google Scholar), encompassing the year range between 2010 and 2020. A systematic review was carried out using the terms such as: landfill leachate, surface and ground water contamination, heavy metals, health risk assessment, developing counties, Bangladesh. Further, the references of those scientific articles were utilized to find other articles. Searching of the related literature and retrieving articles were performed following the PRISMA guideline (Moher et al. 2009; Fakhri et al. 2018). Further, we have assemblage these data to make in depth review on the status of landfill pollution in Bangladesh for the recent decade, how it pollutes the nearby water bodies. In addition, we have analyzed the published dataset to understand leachate pollution level and health risk of leachate toxicity. Although landfill sites are present in every municipalities of Bangladesh, till now studied has been accomplished only on these four landfill sites. Hence, these four landfill sites have been chosen for this review work.

### Study areas

**Fig. 2:**
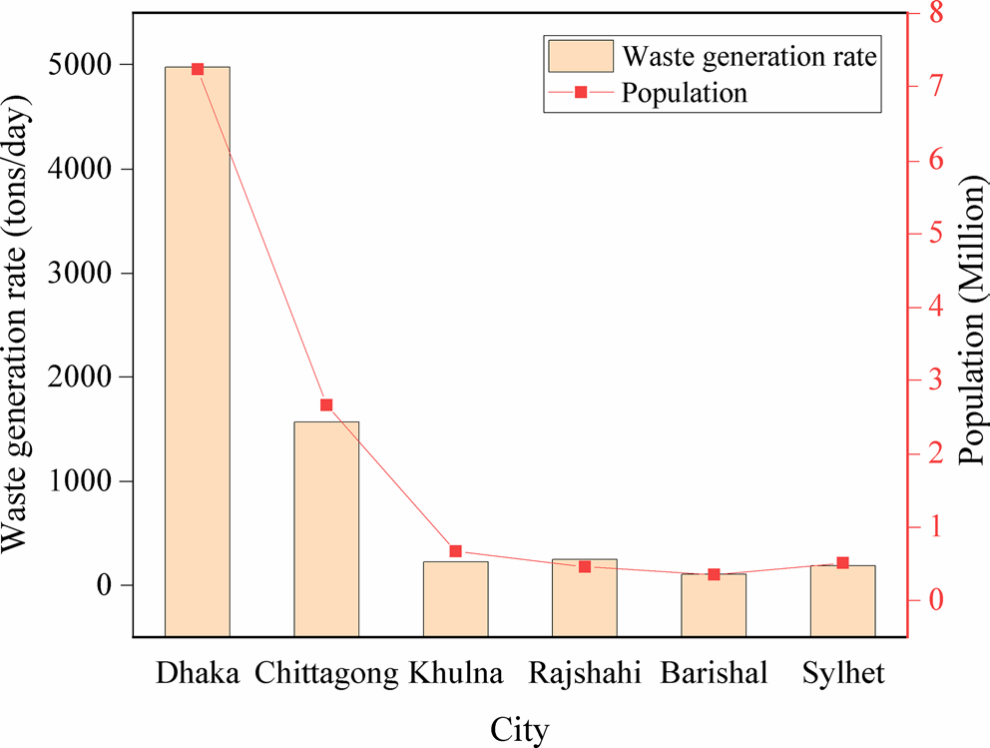
Waste generation rate and population of major cities of Bangladesh. Data has been adapted from (Alam and Qiao 2020)

Dhaka city, the capital of Bangladesh, occupied by 6.73–7.5 million populations, generated around 5000 tons/day (0.56 kg/cap/day) of solid waste in 2005, which may have exceed 30,000 tons/day by 2020 (Fig. 2) (DNCC 2016; Alam and Qiao 2020). The waste generation rate and population of the major cities of Bangladesh are shown in Fig. 2. In Dhaka city, there are two solid waste landfill site, named Amin Bazar and Matuail landfill. The Amin Bazar landfill site (23°47′48″N and 90°17′50″E) is situated in the low-lying floodplain areas of the Karanachhali River in Savar Upazilla, Dhaka (Fig. 1). The area is used as an open dumpsite from 2007 with the total area of about 52 acres (DNCC 2016).

Matuail landfill site (23°43′16.0"N 90°27′01.5"E) is situated in a low-lying agricultural land with the total area of 100 acres (Fig. 1) and more than 60% of total wastes generated daily in this capital are disposed here. This landfill is semi-aerobic which is in pipe system, half circle of it is solid in lower part and upper half is perforated for passing natural air (Jahan et al. 2016; Hossain et al. 2018).

Sylhet city having 0.5 million populations lies in the north-eastern zone of Bangladesh, generated 250 tons/day waste in 2016 (Fig. 2) (Alam and Qiao 2020). Mogla Bazar landfill site (24°51′16.8"N 91°53′23.4″E) is the main landfill site of Sylhet city for solid waste dumping, which is located within the Surma-Kushiyara floodplain in the Mogla Bazar Union under Sylhet Sadar Zilla (Fig. 1). This is an open landfill site with an area of about 10.25 acres. The surface geology of the study area is Alluvial silt and clay, and almost every year this area is inundated with flash flood and (Alam et al. 2020).

With 2.66 million populations, Chittagong city lies in the south eastern zone of Bangladesh, generated around 1161–1548 tons/day of solid waste and average 0.34–0.48 kg/cap/day (Fig. 2) (Abedin and Jahiruddin 2015; Alam and Qiao 2020). Among the two landfill sites of this mega city, Rowfabad landfill (22°18′45.9″N 91°46′22.3″E) is one of them (2.83 acres) (Islam 2016) and very close of the sea shore of bay of Bengal (Fig. 1).

### Data analysis

####  LPI calculation

The leachate pollutant potential of landfill sites of Bangladesh has been calculated (Table 1) using the following leachate pollution index (LPI) Eq. (1), which has been formulated based on the Delphi technique by Kumar and Alappat, (2005).

1 $$ {\text{LPI}} = \mathop \sum \limits_{i = 1}^{n} {\text{w}}_{{\text{i}}} P_{i} /\sum {\text{w}}_{{\text{i}}} $$

where *w*_*i*_ represents the weight factor for the ith pollutant variable, *P*_*i*_ is the sub index score of the ith pollutant variable, and n is the number of known concentrations of leachate contaminant variables (Table 1).

**Table 1:** Leachate characteristics and leachate pollution index (LPI).

Rowfabad landfill					Matuail Landfill				
Serial	Parameters	Conc	Wi	(pi)	(wi*pi)	Conc	Wi	(pi)	(wi*pi)
1	Cr	2	0.064	10	0.64	–	–	–	–
2	Pb	0.027	0.063	5	0.32	0.02	0.063	5	0.32
3	COD	430	0.062	10	0.62	1343	0.062	40	2.48
4	BOD	216	0.061	10	0.61	96	0.061	10	0.61
5	As	0.09	0.061	5	0.305	–	–	–	–
6	Zn	2.5	0.056	5	0.28	2.3	0.056	5	0.28
7	pH	6.5	0.055	5	0.275	8.17	0.055	5	0.275
8	Ni	0	0.052	5	0.26	0.17	0.052	5	0.26
9	Ammonia N	–	–	–	–	980	0.051	100	5.1
10	Cu	0.65	0.05	5	0.25	0.09	0.05	5	0.25
11	TDS	2700	0.05	5	0.25	7120	0.05	20	1
12	Cl	104	0.048	5	0.24	–	–	–	–
13	Fe	7.25	0.045	5	0.225	3.41	0.045	5	0.225
			$\sum {w}_{i}$=0.667		$\sum_{i=1}^{n}{w}_{i}{P}_{i}$=4.27		$\sum {w}_{i}$=0.55		$\sum_{i=1}^{n}{w}_{i}{P}_{i}$=10.80
					LPI = 6.40				LPI = 19.81

All values in mg/L, except pH. Pollutant weight (wi) and Sub-index value (pi) were adapted from (Kumar and Alappat, 2005).

###  Human health risk index for heavy metals in vegetables and fish

Health risk index (HRI) for ingestion of toxic metals through the consumption of vegetables and fish were calculated using daily intake of metals (DIM) (Sridhara Chary et al. 2008; Kortei et al. 2020) and reference oral dose (RfD). The daily intake of metals (DIM) was calculated by the following Eq. (2):

2 $$ {\text{DIM}} = \frac{{C_{{{\text{metal}}}} \times C_{{{\text{factor}}}} \times D_{{\text{food intake}}} }}{{B_{{\text{average weight}}} }} $$

whereC_metal_: heavy metal concentrations in plants (mg kg^−1^).C_factor_: conversion factor (0.085).D_food intake_: daily intake of vegetables.B_average weight_: average body weight.

The average daily vegetable intakes were taken as 0.345 and 0.232 kg/person/day for adults and children respectively (Khan et al. 2008; Kamal et al. 2016), while average daily fish intakes was taken as 0.74 kg/person/day for adults and children (Kortei et al. 2020). The average body weights for adult and children in Bangladesh were taken as 60 and 22 kg, respectively (Khan et al. 2008; Kamal et al. 2016).

The HRI was calculated (Jan et al. 2010) by the following formula:

3 $$ {\text{HRI}} = \frac{{{\text{DIM}}}}{{R_{{{\text{fd}}}} }} $$

where DIM represents the daily intake of metals. *R*_fd_ (reference oral dose) is an estimated per day exposure of metal to the human body that has no detrimental effect during life time. *R*_fd_ value for Pb, Ni, Cu, Cd, Mn, Cr, Fe and Zn is 0.004, 0.02, 0.04, 0.001, 0.033, 1.5, 0.7 and 0.30 (mg/kg bw/day) respectively (USEPA 2015). An HRI value less than 1 is believed to be safe for the exposed human being (Ghosh et al. 2013). HRI for heavy metals in the plant species near Amin Bazar landfill has already been calculated by Kamal et al., 2016 and; for Matuail and Mogla Bazar landfill have been calculated in the present study. The HRI for metal in fish also has been calculated in the present study.

####  Carcinogenic risk

The carcinogenic risks of ingesting the heavy metal containing edible plant (Shaheen et al. 2016), fish (Ahmed et al. 2015) and drinking water (Mohammadi et al. 2019) were evaluated according to the risk assessment guidelines recommended by USEPA. The target carcinogenic risk (CR) factor (lifetime cancer risk corresponding to a specified concentration of a contaminant) (USEPA 1989) can be calculated as

$$ {\text{CR}} = \frac{{\left( {{\text{EF}} \times {\text{ED}} \times {\text{IR}} \times C \times {\text{Csfo}}} \right) \times 10^{ - 3} }}{{\left( {{\text{BW}} \times {\text{AT}}} \right)}} $$

where CR represents the target cancer risk or the risk of cancer over a lifetime, EF is the annual exposure frequency (365 days/year), ED is the exposure duration (70 years for adult in Bangladesh). IR is ingestion rate (g/person/day), which is 130 g/day/person for vegetables, 44.7 for fruits and for rice, 367 g/day/person (Shaheen et al. 2016). For drinking water ingestion, it is considered as 2 L/day/person (ASTDR 2000), e fish ingestion rate 49.5 g/day/person (BBS 2011; Ahmed et al. 2015). C is the metal concentration in food samples (mg/kg) or in drinking water (mg/L). BW is the body weight, which is considered as 60 kg for an adult in Bangladesh) (Heikens 2006). *AT* is the averaging time for non-carcinogens (365 days year-1 × number of exposure years, 70 years), and Csfo is the oral carcinogenic slope factor obtained from the integrated risk information system (USEPA 2015) database, which was 1.5, 0.0085, and 0.38 (mg/kg/day) for As, Pb, and Cd respectively.

## Result and discussion

### Landfill leachate chemical characteristics

Landfill leachate is one of the key anthropogenic heavy metal sources in environment and is a major concern to human health. Till now, open landfilling without segregation is still the most prevalent solid waste dumping method in Bangladesh (DNCC 2016). Hence, it is necessary to get a proper view of leachate contamination in different landfill sites of Bangladesh of the recent decade prior to understand the leachate contamination potential of municipal landfills. The chemical characteristics of leachate of different landfill sites of Bangladesh including heavy metals (Cd, Cr, Cu, Fe, Mn, Ni, Pb, and Zn) and other parameters (BOD, COD, DO, pH, and TDS) are shown in Table 2 and in addition, leachate characteristics of different landfills from South Asian countries (India, Thailand and Malaysia) are also added to make comparison for better understanding the landfill leachate pollution in Bangladesh.

**Table 2:** Comparison of leachate characteristics of different landfill sites in Bangladesh with other studies in south east Asian countries. These values represent the mean value for each site

Area	Zn	Mn	Pb	Cd	Cu	Cr	Fe	Ni	DO	BOD	COD	pH	TDS	Year
*Bangladesh*														
Matuail, Dhaka	0.04	–	BDL	0.005	0.15	0.7	–	1.05	0.9	–	1630	6.9	734	(Azim et al. 2011)
Matuail, Dhaka	–	–	–	BDL	1.5	0.36	25	4.5	–	–	–	7.73	6697	(Aminul Haque et al. 2013)
Matuail, Dhaka	0.07	0.21	0.01	BDL	0.07		3.04		3.64	96	1343	8.17	7120	(Jahan et al. 2016)
Matuail, Dhaka	2.5		0.2		0.2		2.6	1.04	1.34	100	1350	8	7178	(Hossain et al. 2018)
Amin Bazar, Dhaka	–	–	–	0.30	1.14	0.18	14.7	2.32	–	–	–	–	–	(Hoque et al. 2014)
Amin Bazar, Dhaka	–	–	0.45	–	–	–	–	0.33	1.8	244	2090	7.8	5613	(Rikta et al. 2018)
Mogra bazar, Sylhet	0.12	0.88	0.30	0.05	0.16	–	0.65	–	–	–	–	7.9	–	(Alam et al. 2020)
Rowfabad, Chittagong,	2.5	2.12	0.03	0.09	0.65	2	7.25	–	0.8	216	430	6.5	2700	(Hossain et al. 2014)
*South-east Asia*														
Brahmapuram, Kochi, India	0.27	–	0.02	–	0.1	0.12	60	0.53	–	39500	57300	6.6	14490	(Arunbabu et al. 2017)
Ramna MSW, North India	BDL	–	BDL	BDL	0.33	1.77	4.5	BDL	–	1335	8332	8.82	2322	(Mishra et al. 2019)
Pathumthani, Thailand	2.28	–	0.10	0.01	0.11	–	52.7	–	–	418	4300	8.4	18900	(Visvanathan et al. 2007)
Ram Indratransfer station, Bankok, Thailand	0.15	–	0.44	26.5	0.17	–	860	–	–	55880	68500	3.8	20700	(Visvanathan et al. 2007)
AmpangJajar, Malaysia	0.01	–	0.3	–	0	0	3	0	–	48	599	7.5	2543	(Aziz et al. 2010)
Kuala Sepetang, Malaysia	0.2	–	0.4	–	0.75	0.05	5	0.3	–	85	990	8.1	12568	(Aziz et al. 2010)
BerisLalang, Malaysia	1.5	–	1	BDL	5	–	0.06	0.04	0.70	1640	1965	8.31	–	(Fadhullah et al. 2019)

All data are presented here as mg/L, except pH; *BDL* = Below detection limit

Leachate characteristics of those landfill sites are shown in Table 2, demonstrate high variations from site to site and for the same site in different times. This variability might be explained by two different ways. One reason is the leachate composition could be influenced by local conditions, the waste management habits of the residents and landfilling system (Abedin and Jahiruddin 2015; DNCC 2016). Another reason is the concentration of organic pollutants (COD, BOD), TDS and heavy metal declines in the post-monsoon. The organic and inorganic contaminant present in the leachate might be diluted with the precipitations and hence lower concentration of those parameters was observed in post-monsoon season (Mor et al. 2018). For some landfill site, the sample may have been collected during that season and thus get less concentration of COD, BOD, and TDS.

#### Physico-chemical characteristics

The pH of the leachate may varies depending on age of the landfill and concentration of volatile acid due to the presence of methanogenic bacteria (Filho and Miguel 2017). Leachate from young landfill sites has pH varying from 5.0 to 6.5, whereas mature landfill leachate has pH value ranging from 7.8 to 8.64 (Zakaria and Aziz 2018). This finding well corresponds to the pH of fresh leachate and matured leachate in Amin Bazar landfill site of Bangladesh. Rikta et al., (2018), found that the pH of fresh leachate is 5.68 and young and matured leachates are around 8 in Amin Bazar landfill site. As for, Matuail and Mogla Bazar landfill site, the pH value of the leachate found to be 7.8–8 (Table 2) (Aminul Haque et al. 2013; Jahan et al. 2016; Hossain et al. 2018; Alam et al. 2020), indicating those leachate are in intermediate and/or semi-matured stages. Its noteworthy that landfill leachate may enhance the pH of drinking water, and may contribute in trihalomethane (THM) formation which is a toxic substance for human health (Kumar and Alappat, 2005). In contrast to these two landfill sites, the pH was found to be lower (6.5) for Rowfabad landfill site (Hossain et al. 2014), indicating relatively fresh condition.

**Fig. 3:**
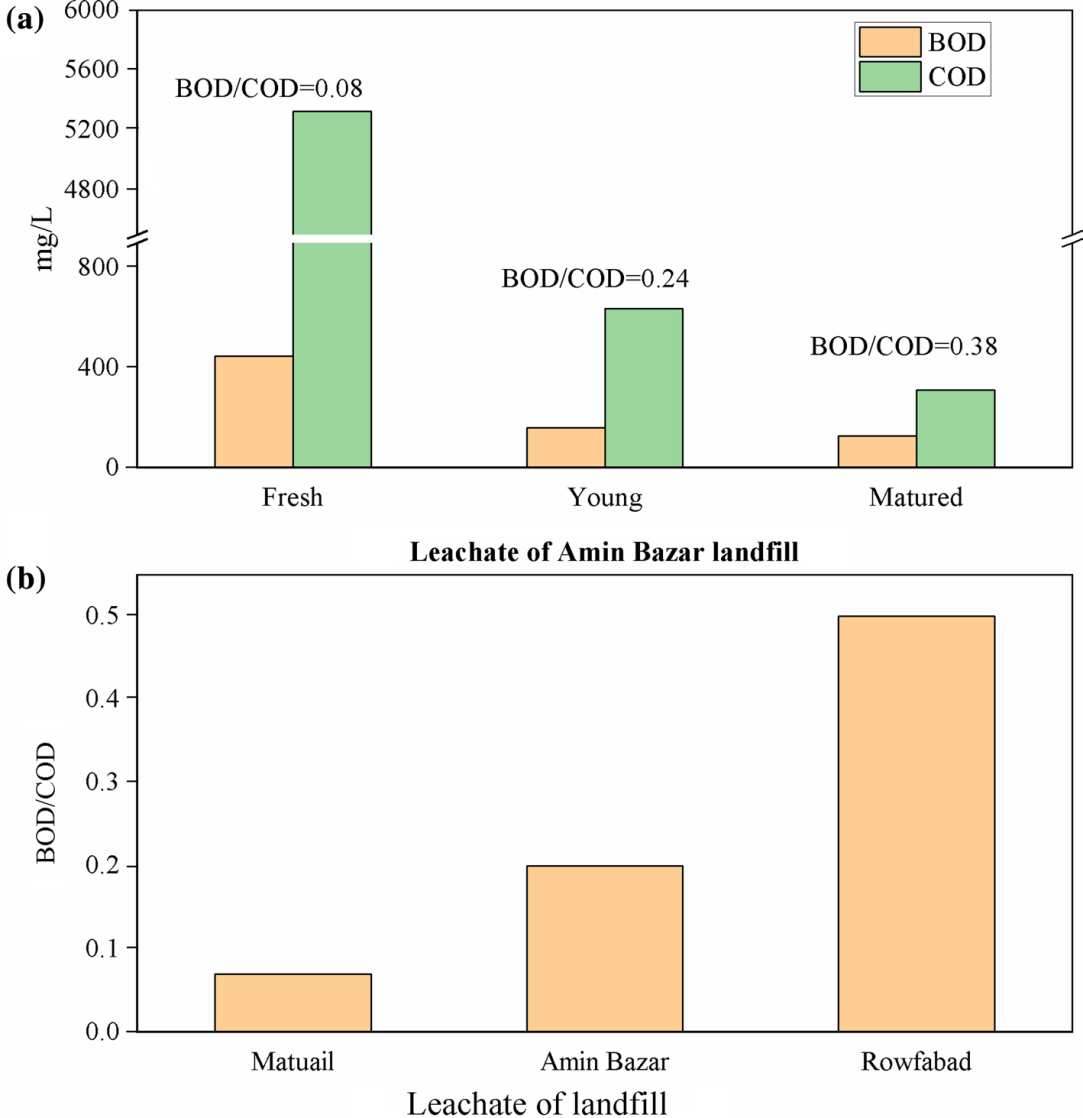
**a** BOD_5_, COD load and BOD_5_/COD ratio of landfill leachate at different ages in Amin Bazar landfill (Rikta et al. 2018); **b** Comparison of BOD_5_/COD ratio of leachate among Matuail (Hossain et al. 2018), Amin Bazar (Rikta et al. 2018), and Rowfabad (Hossain et al. 2014) landfill site

We have calculated the BOD_5_/COD ratios of the four studied landfill site's leachate in Bangladesh (Fig. 3). The BOD_5_/COD ratio of Rowfabad landfill leachate is 0.5 (Hossain et al. 2014) and for Matuail in 2016 is 0.1 (Jahan et al. 2016) and in 2018, it is 0.07 (Hossain et al. 2018) (Fig. 3b). Landfill leachate with BOD_5_/COD ratio less than 0.1 is considered to be toxic, as this level means the presence of a big portions of hardly biodegradable COD (Samudro and Mangkoedihardjo 2010) and this high concentration of COD can play a vital role in modifying the physiochemical properties of groundwater and insert organic contamination in water as reported by different authors (Kaur et al., 2016; Mor et al., 2018). Hence, on the basis of that, the landfill leachate of Matuail can be considered as toxic. In another study on landfill leachate of Amin Bazar, Rikta et al., (2018) reported the BOD and COD of fresh, young and matured leachate, and we calculated the BOD_5_/COD ratio for each state of the leachate. The BOD_5_/COD ratio of fresh leachate is 0.08 and the ratio has increased when the fresh leachate converted to young and finally in matured stage (Fig. 3a). This result again suggests that the fresh leachate is more toxic than the matured one. If this fresh leachate can percolate to the groundwater system or overflowed to the surface water, it may exert high toxic effect on aquatic species and human being, who use that water. Previous studies (Kjeldsen et al. 2002; Gao et al. 2015) also stated that high concentrations of all components in the early acid phase of leachate due to strong decomposition and leaching.

Ammonia-N is one of the major pollutant of leachate as it can persists in the aquatic environment for a long time period and poses threat to both human and aquatic species (Yenigün and Demirel 2013). In Matuail landfill site, high concentration of ammonia-N (980 mg/L) was found (Table 1) (Jahan et al. 2016). Although this level is quite lower in comparison to the concentration of ammonia-N (2240 mg/L) in leachate of Brahmapuram landfill, Kochi, India (Arunbabu et al. 2017). According to (ECR 1997), ammonia-N concentration should not be excess 50 mg/L in inland surface water. Hence this excess ammonia-N containing leachate can affect the water quality seriously, if does not treated properly before discharge.

#### Heavy metals

Heavy metals in leachate have significant impact on groundwater as well as surface water quality even if it found in traces amount in leachate. The leachate samples collected from the landfill sites (Azim et al. 2011; Aminul Haque et al. 2013; Hoque et al. 2014; Jahan et al. 2016; Hossain et al. 2018; Alam et al. 2020) of Bangladesh are enriched in heavy metals, as different types of waste, like batteries, paints containing lead, industrial effluent, plastics and steel pipes are being regularly dumped there without segregation (DNCC 2016). The concentration of toxic metals at the Bangladeshi landfill sites in different year’s ranges from BDL-0.45 mg/L for Pb; BDL-0.30 mg/L for Cd; 0.18–2 mg/L for Cr; 0.07–1.5 mg/L for Cu; and 0.33–4.5 mg/L for Ni (Table 2).

From 2011 to 2018, the leachate of Matuail landfill site was rich in Ni (1.04–4.5 mg/L), Fe (2.6–25 mg/L) and Cr (0.7–0.36 mg/L) and with a lesser concentration of Zn, Mn, Pb, Cd and Cu (Table 2) (Azim et al. 2011; Aminul Haque et al. 2013; Jahan et al. 2016; Hossain et al. 2018). As for, Amin Bazar landfill, the leachate is characterized by high concentrations of Cu, Cr, Fe, Pb and Ni (Hoque et al. 2014; Rikta et al. 2018). Rikta et al., 2018, found high concentration of dissolve organic matter in Amin Bazar landfill leachate and reported that among the different stages of leachate, dissolve organic matter in young leachate has the highest metal (i.e. Ni, Pb, Hg) binding affinity compare to fresh and matured leachate. The Amin Bazar landfill area is flooded with rain water during each monsoon. Hence, during the monsoon/post monsoon, there is a chance of surface water pollution from this landfill site to the nearby villages (Konda, Baliarpur), which are situated within a distance of 1 km from the landfill site (Kamal et al. 2016) and densely populated as a sub-urban areas of Dhaka.

Further, the concentration of Pb is found to be high (0.16 mg/L) in leachate of Mogla Bazar landfill site (Alam et al. 2020) and the concentration of Zn, Mn, Cu and Fe is high in Rowfabad landfill (Hossain et al. 2014). Alampur and Shunar Gaon Union are situated just beside the Mogla Bazar landfill site (Alam et al. 2020) and these areas are highly vulnerable to flash flood. Hence, there is a high possibility of mix-up of the toxic leachate with surface water during monsoon in those areas. In addition, most of the solid waste landfill sites in Bangladesh were installed in the low lying areas without any feasibility study and proper lining is barely exercised at those dumping sites. However, metal contamination, as well as BOD, COD load in the leachate of Mogla Bazar landfill site, Sylhet and Rowfabad, Chittagong are lower compare to those of Dhaka city (Table 2). Further, the waste generation rate and population are also lower in Chittagong and Sylhet compare to those in Dhaka (Fig. 2 and Table 2). This finding suggests that the landfill leachate pollution in a city may proportional to the number of population and waste generation rates.

The leachate characteristics of different landfills of Bangladesh are comparable with those leachate of Ramna solid waste landfill, North India (Mishra et al. 2019) and; Beris Lalang (Fadhullah et al. 2019), Ampang Jajar and Kuala Sepetang (Aziz et al. 2010) landfill sites of Malaysia. However, the level of contamination, especially the organic one in the leachate of the landfills of Bangladesh are quite lower compare to Brahmapuram, Kochi, India (Arunbabu et al. 2017), Pathumthani and Ram Indra transfer station Landfill, Thailand (Visvanathan et al. 2007). The reason behind the lower value of BOD, COD and TDS of the Bangladeshi landfill sites compared to the landfill sites of other countries (Table 2) are: (1) the samples from Bangladeshi landfill sites has collected from the pond, where the leachate has already reached the mature stage; and (2) because of the lack of proper liner system in Bangladeshi landfill sites, the decomposed liquid organic waste has percolated to the underlying soil, which lowered the BOD, COD and TDS of the leachate.

#### Prediction of the occurrence of xenobiotic/emerging contaminants and micro-plastic

Landfill sites can be a source of several organic chemicals (as landfill leachate) including pesticides (Kibria et al. 2010), pharmaceuticals (Mompelat et al. 2009), plastic additive chemicals (DEHP, PBDEs) (Meeker et al. 2009), and per fluorinated substances (PFOS, PFOA) (Gallen et al. 2017). Such chemicals can contaminate both surface and groundwater close to landfills.

Xenobiotic organic compounds are originated in the landfill leachate from household or industrial chemicals and exists in comparatively low concentrations (generally less than 1 μg/L). Hence, very advance analytical instruments are needed to quantify these compounds. These compounds include a variety of aromatic hydrocarbons, pesticides, chlorinated aliphatics, phenols, and plasticizers (Kjeldsen et al. 2002). Due to lack of advance analytical tools and complexities, no studies have been accomplished on the occurrence and fate of xenobiotic/emerging contaminants and micro-plastic generated from the landfill leachate of Bangladesh. However, we can infer the occurrence of these contaminants in the landfill site of Bangladesh and its transport in the water stream, as the composition of dumped solid waste in Bangladeshi landfill is known (Alam and Qiao 2020).

Now-a-days, it is possible to investigate the fate of unused and/or expired pharmaceutical and personal care products (PPCP) that many countries are usually discarded in municipal solid waste (Yi et al. 2017; Borquaye et al. 2019; Yu et al. 2020). When these PPCP wastes are dumped into a landfill, microorganisms in the landfill are exposed to the medicines, specially antibiotics, turns bacteria into lethal mercenaries and diseases caused by them may become incurable in near future. Yi et al., (2017) provides information on the occurrence of many emerging contaminants and antibiotic resistance genes in raw leachate from 16-year old closed landfill site in Singapore. In the very recent study in China, it has been found that the composition of PPCPs in groundwater are matched to that in raw landfill leachate (Yu et al. 2020). According to many research works, landfill leachate is an important source of emerging contaminants, i.e. PPCP waste and antibiotic resistance genes into the environment which might pose a threat to ground and surface water in the vicinity of landfill site. As in Bangladesh, the medical wastes are also dumped as solid waste in the landfill (Hossain and Alam 2013), it is inferred to have considerable concentration of emerging contaminant and antibiotic residues in the leachate and thereby migration in the surface and ground water. And, these may pose a detrimental threat to public health of the densely populated Bangladesh. Therefore, identifying the occurrences and fate of antibiotic residues in landfill leachate and surrounding water bodies are decisive.

Conversely, recent studies (Su et al. 2019; He et al. 2019) provided evidence of microplastic in landfill leachate (around 13 items/L) in China and He et al., (2019) stated that landfill may not the final sink of plastics, rather a potential source of microplastics; which also act as a vector for different micropolllutants (Bollmann et al. 2019). Further, Urase et al., (2007) found micropollutants (bisphenol and toluene) in leachate from an open dump site in Thailand. In the solid waste disposal site, heat is generated by the biodegradation of wastes and this play a role in the release of the micropollutants from plastic wastes (Urase et al. 2007). These microplastic and micropollutants from leachate can enter in aquatic body through surface runoff and thereby can ingested and accumulated in different tissues of fish, lead to perturbations in fish biological systems (Ding et al. 2018). In this consequence, if those fishes are consumed by human, it will further bioaccumulate in human. As for Bangladesh, among the different categories of waste that are dump in solid waste landfill, on an average around 4% of it comprises polythene and plastic (Alam and Qiao 2020). In the very recent time, soil samples were collected from the Amin bazar landfill sites and the presence of microplastic has been identified in the form of low density polyethylene (LDPE), high density polyethylene (HDPE), and cellulose acetate (CA) (Afrin et al. 2020). Hence, we can hypothesize the possibility of release microplastic and micropollutants from the landfill leachate of the dumping site in Bangladesh to the receiving water body and thereby bioaccumulation in aquatic species (Parvin et al. 2021). Future research is required to identify the occurrence of these emerging contaminants in landfill leachate and in the surface water in the vicinity of the landfill site.

### Leachate pollution Index of different landfill sites

Leachate characteristics demonstrate high variations from site to site and different parameter's concentration may vary over several order of magnitude (Hossain et al. 2014; Jahan et al. 2016; Arunbabu et al. 2017; Mishra et al. 2019). Thus it is important to use quantitative tool to compare the leachate pollution potential among different municipal landfills. Kumar and Alappat, (2005) developed an index known as LPI, for quantifying the leachate contamination potential of municipal landfills conveniently. This is a quantitative tool for summarizing complex leachate pollution data of landfill sites uniformly. Furthermore, it is interesting to note that, the phytotoxic effects of leachate in plants can also be understood by the LPI values. Arunbabu et al., (2017) reported that leachate having LPI values larger than 10 are likely to exert phytotoxic effects on plant.

**Fig. 4:**
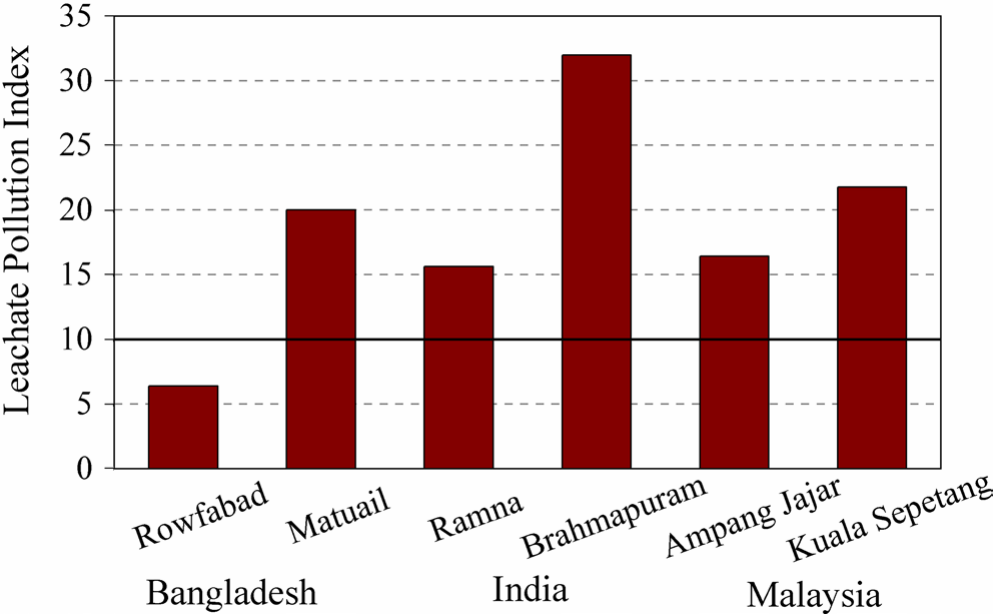
LPI value of Rowfabad landfill in Chittagong, Matuail landfill in Dhaka and their comparison with LPI value of the landfill sites of India, named Brahmapuram (Kochi), Kerala (Arunbabu et al. 2017); Ramna (Mishra et al. 2019) and of Malaysia named Ampang Jajar Landfill; Kuala Sepetang Landfill Site (Aziz et al. 2010). LPI values greater than 10 are likely to exert detrimental effect on environment and human health

LPI of Rowfabad and Matuail landfill site was estimated using the published data (Hossain et al. 2014; Jahan et al. 2016), which is shown in Table 1. However, we could not calculate the LPI of Amin Bazar and Mogla Bazar landfill site, as the authors (Rikta et al. 2018; Alam et al. 2020) has studied few parameters of the leachate, by which it is difficult to estimate the LPI value without error. The LPI value of the Bangladeshi landfill sites have been compared with that of the landfill sites of India and Malaysia, prior to understand the leachate contamination status of Bangladesh compare to the neighboring country, which is shown in Fig. 4.

LPI of Rowfabad landfill site (6.4) is very low compared to that of Matuail landfill (19.9), indicating leachate contamination potential of Rowfabad landfill site is comparatively less (Fig. 4). Very high value of COD and TDS in the leachate of Matuail landfill compared to Rowfabad landfill (Table 1) makes the LPI of value for Matuail landfill site higher. On the other hand, the high LPI value of Matuail landfill site indicates that leachate generated from that landfill site may not stabilized and mature enough, and thus have comparatively high risks of contaminating the groundwater of nearby areas (Naveen et al. 2018). The high value of LPI (> 10) indicates a hazardous nature of the landfill and has the potential to contaminate surrounding groundwater (Mor et al. 2018; Mishra et al. 2019). The LPI value of Matuail landfill site (19.9) is higher in comparison to the Ramna landfill site, India (15.6) (Mishra et al. 2019) and semiaerobic Ampang Jajar, Malaysia (16.4) (Aziz et al. 2010), comparable to improved anaerobic Kuala Sepetang landfill Site, Malaysia (21.0) (Aziz et al. 2010), but quite less in comparison to that of Brahmapuram (Kochi), Kerala, India (31.9) (Arunbabu et al. 2017). The reason behind the very high LPI value of Brahmapuram landfill is having extremely high concentration of BOD, COD and TDS (Table 2). Whereas, the concentration of BOD, COD and TDS are comparatively very lower in the leachate of Matuail landfill, which makes the LPI value of that landfill lower compare to Brahmapuram landfill.

### Leachate heavy metal mobilization and water pollution

**Fig. 5:**
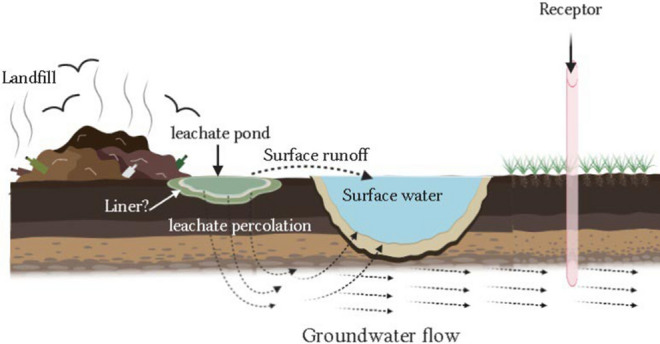
Illustration of landfill leachate (with improper lining/no lining) mobilization in groundwater and surface water

The most common pathway for leachate to mobilize to the aquatic environments is from the bottom of the landfill through the unsaturated soil layers to the ground water (Fadhullah et al. 2019; Mishra et al. 2019). Since most of the landfills in developing countries, especially in Bangladesh, were constructed without engineered liners and proper leachate collection systems (Kjeldsen et al. 2002; DNCC 2016; Alam et al. 2020), leachate could be moved from groundwater to surface water through hydraulic connections. During monsoon floods in sub-tropical country like Bangladesh, it might be directly added to surface water due to fragile construction of landfill site as well as poor managements. The processes by which the toxic leachate from the leachate pond having improper lining/no lining, mobilize into groundwater and surface water have been illustrated in Fig. 5. Several physical, chemical and biological factors might be involved with the leachate migration resulting in modification of the composition and reduction of strength from the original. These migration factors might be depend on the soil stratification beneath the landfill, the hydraulic properties of the ground water system, and the chemical composition of the leachate. The mobilization process may reduce the toxicity of leachate and its potential impact on groundwater and surface water (Naveen et al. 2018). Assessment of heavy metals and organic contamination in surface and groundwater near the landfill sites of developing country is decisive, as aquatic plants and animals reside in this area. And even local people use that ground water for drinking regularly; thus, they are at risk of heavy metal exposure. The physico-chemical properties of the surface and ground water in the vicinity of the landfill sites in Bangladesh are shown in Table 3.

**Table 3:** Physico-chemical characteristics of surface water and ground water in the vicinity of the landfill sites in Bangladesh

Landfill sites	Zn	Mn	Pb	Cd	Cu	Cr	Fe	Ni	DO	BOD	COD	pH	TDS	References
*Surface water*														
Matuail	0.116	–	0.03	0.005	0.2	1.03	–	0.4	2.3	–	50	6.5	427	(Azim et al. 2011)
Matuail	–	–	0.01	BDL	0.07	–	3.04	0.09	3.64	49	328	8.2	3360	(Jahan et al. 2016)
Mogra Bazar	0.01	0.03	0.33	0.00	0.01	–	0.03	–	–	–	–	6.3	–	(Alam et al. 2020)
Rowfabad	0.14	0.015	0.005	0.067	0.25	1.07	4.8	–	2.35	21	69	7.2	–	(Hossain et al. 2014)
Inland surface water standard	5	5	0.10	0.05	0.5	0.1	2	1	4.5–8	50	200	6–9	2100	(ECR, 1997)
*Ground water*														
Matuail	5	–	0.05	0.001	0.01	0.05		0.02			3.88	6.8	502	(Azim et al. 2011)
Mogra Bazar	0.02	–	0.17	–	0.02	–	0.04	–				7.1		(Alam et al. 2020)
Rowfabad	0.5	0.12	0.007	0.04	0.02	0.09	3.26		2.45	0.4	44	6.7		(Hossain et al. 2014)
WHO guideline for Drinking water	3	0.2	0.01	0.003	2	0.05	0.3	0.02	NM	NM	NM	6.5–9.2	500	(WHO, 2012)
Standards for drinking water	5	0.1	0.05	0.005	1	0.05	0.3–1	0.1	6	0.2	4	6.5–8.5	1000	(ECR, 1997)

All values are in mg/l, except pH

Azim et al., (2011) studied the surface and ground water at the vicinity of the Matuail landfill sites and reported that most of the water quality parameters of the surface water within 1 km radius from Matuail landfill were below the safe limit of inland surface water (ECR, 1997), with the exclusion of Cr (1.03 mg/L) and DO (2.3 mg/L). Further, Jahan et al., (2016) studied the surface water near the same landfill site, and reported that the concentration of Fe, TDS, COD load and DO did not comply with inland surface water standard (ECR 1997) (Table 3). Oxygen depletion in the surface water is considered as a main potential effect of leachate discharge to water bodies, as the decrease of DO can affect the stream bottom fauna, flora and generate ammonia toxicity (Kjeldsen et al. 2002). Conversely, the lowlands surrounding the Matuail landfill site (Fig. 1) are used for fisheries. Hence, there is a possibility of bioaccumulation of those heavy metal in fish and thereby to humans. However, the concentration of metal ion in groundwater near the Matuail landfill site (Azim et al. 2011) was found to be generally very low due to the decreasing solubility of metal, although the Zn and Pb concentration exceeded the standard value for drinking water set by WHO (WHO 2012). However, with the increasing burden of solid waste in those landfill site (Alam and Qiao 2020), level of pollution is predicted to increased. As for Amin Bazar landfill site, in spite of having poor waste management and leachate treatment, location in the residential, as well as designated flood flow zone area, unfortunately, till date best of our knowledges, no study has been found on surface and groundwater contamination near the landfill site.

As for, Mogla Bazar landfill site, Alam et al., (2020) reported that all the toxic metal's concentration in the surface and ground water in the vicinity of the landfill site, are below the safe limit for inland surface water and drinking water set by DoE, Bangladesh and WHO (ECR 1997; WHO 2012), except for Pb (0.33 mg/L in surface water and 0.17 mg/L in groundwater). Special attention should be taken for preventing the mobilization of the toxic heavy metal to ground water, as this water is used by the nearby resident for drinking purposes.

Further, Hossain et al., (2014) reported that the surface water in the vicinity of Rowfabad landfill site contain high concentration of Fe, Cr, and Cd (Table 3). The concentration of Fe, Cd and Cr in the groundwater near the Rowfabad landfill (Hossain et al. 2014) are found to exceed the maximum permissible limit (MPL) for drinking water (ECR 1997; WHO 2012). Further, Hossain et al., (2014) found that the presence of fecal indicator bacterium E. coli in ground water samples (15/100 mL in winter and 71/100 mL in rainy season). Faecal coliform provides an indication of the potential for contribution of enteric bacteria pathogens from the various sources of solid waste (Gerba et al. 2011). Hence, it is predicted to have serious health implications of the residents, who drink water from those groundwater sources. However, the BOD, COD load and TDS of the groundwater in vicinity of Matuail (Azim et al. 2011) and Rowfabad (Hossain et al. 2014) landfill are below and/or very close to the MPL of drinking water, indicating lower chance of organic contamination from the leachate to the groundwater of surrounding site.

### Risk of landfill leachate

**Fig. 6:**
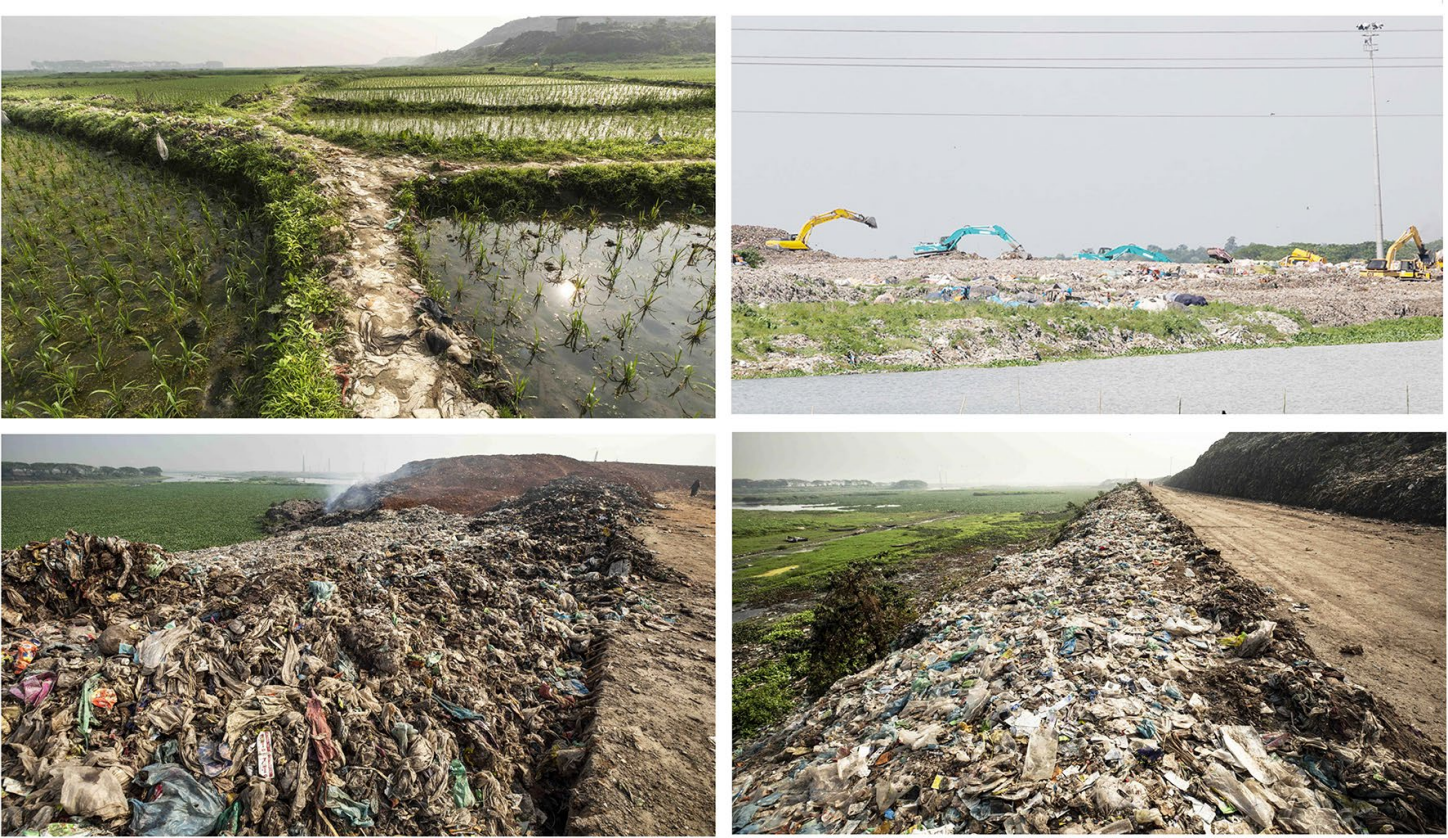
Pictures showing the proximity of the agricultural land and water body to the Amin Bazar landfill site. Pictures are adapted from (https://www.dhakatribune.com/bangladesh/dhaka/2019/08/29/a-landfill-in-flood-flow-zone and https://tbsnews.net/environment/aminbazar-landfill-ruined-lives-54643)

Most of the landfill sites in Bangladesh are situated at designated flood flow zone, specially Amin Bazar and Mogla Bazar landfill (Kamal et al. 2016; Alam et al. 2020). In each monsoon, those areas are reported to inundate, which may result in mix-up of the toxic leachate with surface water and with nearby agricultural soil. In addition, the residents who live near the landfill areas alleged that the city corporations are discharging untreated leachate onto the privately owned land agriculture land. Figure 6 shows the proximity of the agricultural field and water body to the Amin Bazar landfill site. Hence, the toxic heavy metals of the leachate are easily translocated in the nearby water body and soil. Consequently, this heavy metal can bioaccumulate in the agricultural crops which grown in those areas and serious human health problems can be developed by in taking these dietary heavy metal through food crops irrigated and/or flooded with this contaminated water (Mahmood and Malik 2014). Conversely, heavy metal as well as microplastic might be bioaccumulate in fish and other aquatic organisms which lived in those water bodies. A number of heavy metals are highly bioaccumulative and can be adsorbed by microplastics found in landfills. Furthermore, microplastics can be mistaken as food/plankton by fish and other aquatic biota. Therefore, there is possible of transfer of heavy metals to humans via the food chain (from consumption of fish).

####  Assessment of health risk

**Fig. 7:**
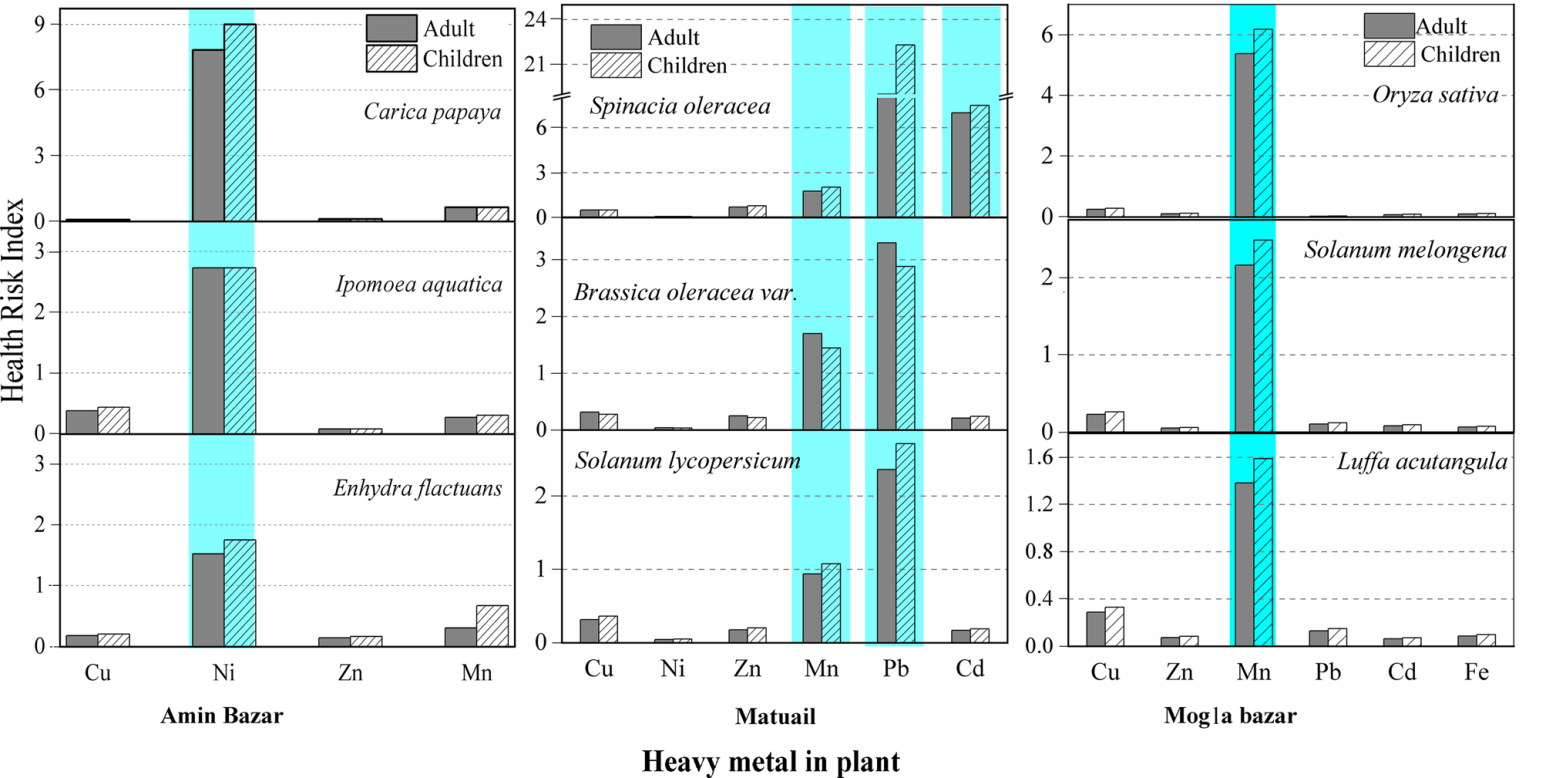
Human health risk index (HRI) for heavy metal in different plant species grown in the vicinity of Amin Bazar, Matuail and Mogla Bazar landfill sites. The blue shaded area represents the HRI > 1, for different heavy metals in plants grown around these landfill sites. The concentration of heavy metals in the plants were adopted from Jahan et al. 2016; Kamal et al. 2016; Alam et al. 2020 adapted from the previous works

Jahan et al., (2016) collected plant species from 3 different field sites around the Matuail landfill and analyzed the concentration of heavy metal in plants, named (*Spinacia oleracea, Brassica oleracea and Solanum lycopersicum*). We have calculated HRI (both adult and children) for heavy metals by consumption of vegetables (Fig. 7). The HRI for Pb, Cd and Mn in both children and adult for these three plant species are found greater than 1 and specially for Pb, all the plant show very high HRI (Pb: 2.36–22). Exposure to Pb through food may cause anemia, weakness, and kidney and brain damage, especially children are more susceptible than adults, while Cd is a highly toxic carcinogenic that is harmful to most of the body’s systems (Hutton 1987; Järup and Åkesson 2009; Jaishankar et al. 2014). Hence, serious health complication is expected in the people who will consume these vegetables. Among these three plant species *Spinacia* *oleracea* shows very HRI for Pb and Cd. Another study (Mahmood and Malik 2014) assessed the HRI of heavy metals via consumption of contaminated vegetables in Pakistan and found that *Spinacia* *oleracea* shows very high HRI of heavy metals, indicating this leafy vegetable has a higher capability to accumulate the heavy metals from soil compared with the others.

Kamal et al., (2016) studied the accumulation of heavy metal in vegetables (*Carica papaya, Ipomoea aquatica, Enhydra flactuans*) from the agricultural fields near Amin Bazar landfill. For those plants, HRI value > 1 was found for Ni (1.76–9), which is both neurotoxic and carcinogenic for human (Genchi et al. 2020). The trend of Ni accumulation in plant species were found in the order of *Carica papaya* > *Ipomoea aquatica* > *Enhydra flactuans* (Kamal et al. 2016). Apart from this human health effect, Ni exert different toxic effects on plants too, which include the plant growth, as well as alterations in the germination process (Nagajyoti et al. 2010). Although metal toxicity restricts the growth of plant roots, stems and leaves, some plants are tolerant to toxic metals (Pollard 2016).

*Oriza Sativa, Solanum Melongena and Luffa Acutangula* grown in the agricultural field around this Mogla Bazar landfill site, show HRI > 1 for Mn (1.38–6.18) (Fig. 7). Mn is both an essential nutrient and a potential neurotoxicant. Excess uptake of manganese through food by humans can may cause dopaminergic dysfunction (O’Neal and Zheng 2015). Among these three plant species; *Oriza Sativa* (rice) shows very high HRI for Mn (5.67–6.18) and might be ahyperaccumulator of several heavy metals. Rice grain is the staple food of Bangladesh, which people consumed almost everyday (0.45 kg/day) and it is cultivated in everywhere of our country. Hence, consuming a cereal food contaning considerable amount of heavy metal, almost everyday, can cause serious health problem of human.

**Fig. 8:**
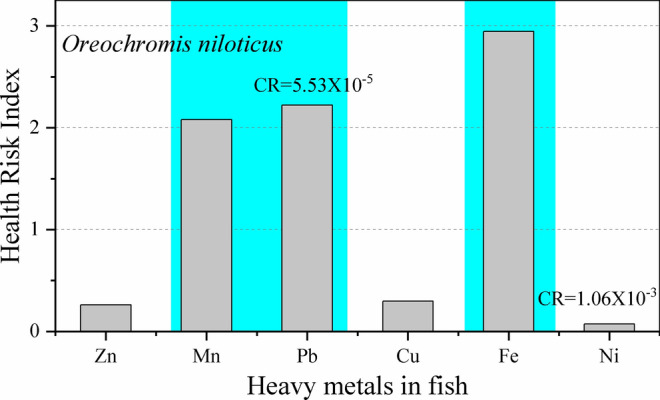
Human health risk index for heavy metals in Tilapia (*Oreochromis niloticus*) fish cultured in the nearby water-body beside Matuail landfill site. The lifetime cancer risk (CR) values for Ni and Pb through the consumption of this fish are shown inside the figure

Hossain et al., (2018) studied the heavy metal contamination in Tilapia (*Oreochromis niloticus*) fish that was cultured in the nearby water-body beside Matuail landfill site and found high concentration of different toxic metals in fish. The calculated health risk (Fig. 8) from heavy metal exposure through this Tilapia fish consumption showed HRI is greater than 2 for Fe (2.9), Pb (2.2) and Mn (2.07). From this result, it can be inferred that serious health complications can be occurred in the residents who will consume this fish those are cultured in the water bodies near the landfill sites.

####  Assessment of cancer risk:

**Fig. 9:**
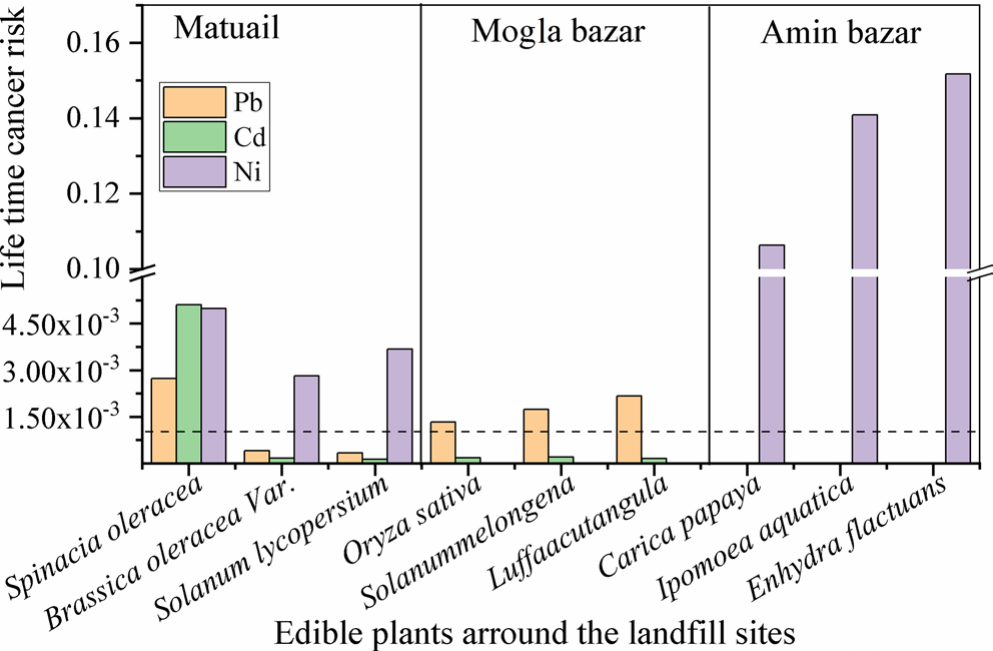
Life time cancer risk (CR) for Ni, Cd and Pb through the consumption of vegetables, fruits and rice grains, grown near Matuail, Amin Bazar and Mogla Bazar landfill sites

The life time cancer risk (CR) for Pb, Cd, and Ni intake through the consumption of vegetables, fish and drinking water (ground water) were calculated (Figs. 8, 9 and table S2), as these metals are classified as carcinogens by the International Agency for Research on Cancer (Kim et al. 2015). Cancer risks will be considered “essentially negligible” where the estimated CR is ≤ 1 × 10^–4^ (USEPA 1989, 2015). If the CR is greater than 1 × 10^–4^, risk can exists by ingesting those foods and water. The CR value for Ni ranges from 1.06 × 10^–1^ to 2.8 × 10^–3^ in vegetables grown near Amin Bazar and Matuail Landfill site. CRs for Pb ranges from 3.23 × 10^–4^ to 8.7 × 10^–3^ in vegetables and rice grain grown near Matuail and Mogla Bazar landfill site. The CR values are found within 2.73 × 10^–3^ to 5.1 × 10^–3^ for Ni, Cd, and Pb by the consumption of *Spinacia* *oleracea* which was grown near Matuail landfill site. In Tilapia fish, cultured in the water body near the Matuail landfill, the CR value for Ni is 1.06 × 10^−3.^ Hence, potential concern exists for Pb and Ni induced CR, by consuming fish and vegetables, grown near the landfill sites. However, as for the ground water near those landfill sites, CRs of Pb, Cd and Ni are found to be lower than the negligible range, except for Cd (1.33 × 10^–3^) near Rowfabad landfill site (Table S2).

### Mitigation options and future challenges

Landfill leachate of different sites of Bangladesh polluted both surface and ground water and it possess serious threat to public health through food chain. Hence, it is necessary to develop necessary mitigation options for landfill leachate pollution. The first approach for preventing the leachate pollution is segregating the waste at sources or dumping site before disposal. If the landfill site is properly controlled by segregating the waste at their source, then the different sort of waste could be managed in different ways, like composting for organic waste; recycling for electronic, paper, and plastic waste; and solidification/stabilization for the hazardous waste. In solidification and stabilization process, specialized additives or reagents are mixed with the hazardous waste materials to reduce the solubility or mobility of contaminants in the surrounding environmental matrix (Ioannidis and Zouboulis 2005). In Bangladesh, decomposed solid waste can be reused as construction material after proper solidification/stabilization.

In spite of having adverse impact on environment, we cannot avoid the waste dumping and land filling methods, as developing countries like Bangladesh land filling is the most convenient way of waste management. In this case, landfills should be constructed with synthetic membranes and/or other possible engineering materials to prevent heavy metal and others toxic materials from escaping into soil and groundwater. In addition, the generated leachate can be drained through pipes into a sewer system where they can be retained, incinerated or further treated. As for, all the existing open landfills with improper lining systems in Bangladesh, there is a mountain of waste, with a height of 50–70 feet high (DNCC 2016; Islam 2016). The major future challenge will be managing the fate of those waste, which include the mixtures of organic waste, plastic, paper, glass, hazardous waste such as paint, batteries and cleaning solvent, medical waste including personnel protective equipment (PPE) using due to COVID-19 and e-wastes.

## Conclusions

This is the first holistic approaches to have characterized the landfill leachate from different mega cities of Bangladesh and how this toxic leachate mobilizes in the ground and surface water. Unlike the other south-east Asian country's landfill, the leachate in Bangladeshi landfills shows high inorganic contamination rather than organic contamination. Our study suggests that the landfill leachate pollution in a city may proportional to the number of population and waste generation rate of a city. These landfill's leachate are found to contaminate the surface and ground water, because of the absence of lining system in the leachate pond, improper treatment and surface runoff due monsoon floods. The presence of toxic heavy metal in the groundwater around those landfill sites does not favour to drinking as per the WHO and DoE standard, especially near the Matuail and Rowfabad landfill site. Further, the generation and migration of the toxic leachate from those landfill sites exert impact on agriculture products. High HRI for Pb, Cd, Ni and Mn in the edible plant and rice grain around these landfill sites were found, especially in *Spinacia oleracea, Carica papaya* and *Oriza Sativa.* Concerning the CR, the total CRs of Ni and Pb were found to be very high in vegetables, suggesting potential concern for Pb and Ni-induced CR through consumption of the studied vegetables and grain. The findings of this study is a ‘wake-up’ call for the policy makers in developing countries for improving solid waste management ansd landfilling to protect the water streams from landfill site pollution and also to reduce the human health risk. One limitation of this study is that the data on leachate pollution and physicochemical characters of surface and ground water of Bangladesh are not yet sufficient, for in-depth review and comparison. Further work on recent status of landfill sites pollution and its impact on water body for all sites is vital to constructing a more holistic overview on landfill leachate contamination in Bangladesh.

## Supplementary Information

Below is the link to the electronic supplementary material.

Supplementary file1 (DOCX 1060 KB)
