# Supplementary material for: Impact of landfill leachate contamination on surface and groundwater of Bangladesh: a systematic review and possible public health risks assessment
Source: Appl Water Sci. 2021 May 29;11(6):100. doi: 10.1007/s13201-021-01431-3 (PMC8164486; doi:10.1007/s13201-021-01431-3)
Supplement: Supplementary file 1 — Supplementary file1 (DOCX 1060 KB) [file 13201_2021_1431_MOESM1_ESM.docx]

**Supporting material for**

**Impact of landfill leachate contamination on freshwater and groundwater of Bangladesh: a systematic review and possible public health risks assessment**

**Fahmida Parvin^a^, Shafi M Tareq^a^**

*^a^Department of Environmental Sciences, Jahangirnagar University, Savar, Dhaka,-1342 Bangladesh*

**Corresponding author:** Fahmida Parvin, Email: fahmidaprvn497@juniv.edu

Research highlights

- Leachate contamination potential of four major landfills of Bangladesh has assessed
- Leachate pollution index for Matuail landfill of Bangladesh showed high value
- Effects of leachate leakage on water body as well as on human health has appraised
- Surface and ground water of the landfill sites have high concentration of toxic metals
- Edible plants of landfill sites demonstrated high carcinogenic risk for Ni and Pb


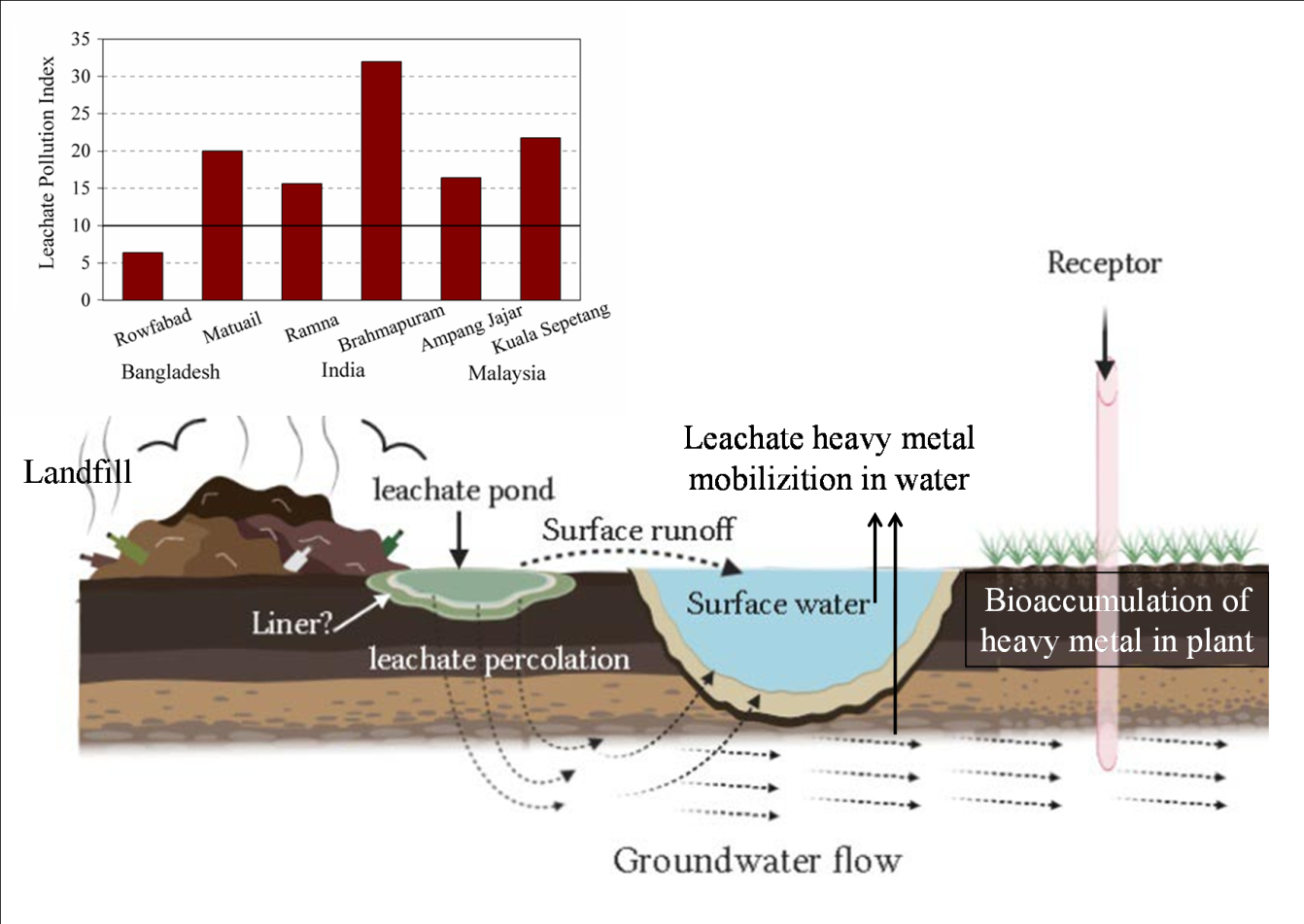


Figure: Graphical abstract

**Table S1:** Lifetime cancer risk for toxic metals through drinking water collected from the ground water near the landfill sites of Bangladesh

| **Landfill sites** | **Life time cancer risk** | | | Reference of the concentration data |
| --- | --- | --- | --- | --- |
|  | **Pb** | **Cd** | **Ni** |  |
| **Ground water** |  |  |  |  |
| Rowfabad | 1.98E-06 | **1.33E-03** |  | Hossain et al., 2014 |
| Matuail | 1.42E-05 | 3.33E-05 | 6.67E-04 | Azim et al., 2011 |
| Mogra Bazar | 4.82E-05 |  |  | Alam et al., 2020 |
